# Characterization and effectiveness of a Fracture Liaison Services program in Colombia

**DOI:** 10.1007/s11657-023-01331-w

**Published:** 2023-10-03

**Authors:** Luis Fernando Valladales-Restrepo, Edgar Eduardo Castro-Osorio, Jessica Ramírez-Osorio, Luisa Fernanda Echeverry-Martinez, Verónica Sánchez-Ríos, Andrés Gaviria-Mendoza, Manuel Enrique Machado-Duque, Jorge Enrique Machado-Alba

**Affiliations:** 1 Grupo de Investigación en Farmacoepidemiología y Farmacovigilancia, Universidad Tecnológica de Pereira-Audifarma S.A, Calle 105 # 14-140, Pereira, Risaralda Colombia; 2 Grupo de Investigación Biomedicina, Facultad de Medicina, Fundación Universitaria Autónoma de Las Américas, Pereira, Colombia; 3 Semillero de Investigación en Farmacología Geriátrica, Grupo de Investigación Biomedicina, Facultad de Medicina, Fundación Universitaria Autónoma de Las Américas, Pereira, Risaralda Colombia; 4 SES Hospital Universitario de Caldas, Manizales, Colombia

**Keywords:** Denosumab, Fractures, Bone, Osteoporosis, Falls, Preventive Health Services, Teriparatide

## Abstract

**Purpose:**

Fracture Liaison Services programs reduce mortality and the risk of refracture and increase treatment and adherence rates. Greater coverage is an important priority for the future. The aim was to determine the characteristics of patients over 50 years old who suffered fractures and the effectiveness of a Fracture Liaison Services program in a health care institution in Colombia.

**Methods:**

This was a retrospective follow-up study of a cohort of patients with vertebral and nonvertebral fractures managed in a Fracture Liaison Services program. Sociodemographic, clinical and pharmacological variables were identified. Key performance indicators were used to evaluate the effectiveness of the program. Descriptive and bivariate analysis was performed.

**Results:**

A total of 438 patients were analyzed. The average age was 77.5 years, and 78.5% were women. Hip and vertebral fractures were the most common (25.3% and 24.9%, respectively). Vertebral fractures prevailed in men (33.0% vs 22.7%; *p* = 0.041) and those of the radius/ulna in women (20.3% vs 10.6%; *p* = 0.031). A total of 29.7% had experienced a previous fracture, and 16.7% had received antiosteoporosis drugs. A total of 63.5% of the cases were managed surgically. At discharge, 58.8% received prescriptions for calcium/vitamin D, and 50.7% with prescriptions of antiosteoporotic therapy, especially teriparatide (21.2%) and denosumab (16.4%), without significant differences by sex. However, in women with hip fractures, anti-osteoporotic management prevailed (83.7% vs 64.0; *p* = 0.032). The effectiveness of the overall program per year was 74.6%. On follow-up, only 9.1% of patients had experienced a new fall, and of those 3.7% presented a new fracture. A total of 4.3% died during follow-up.

**Conclusions:**

Good adherence to the recommendations of the country's clinical practice guidelines was found, and overall, the effectiveness of the program was very satisfactory, with a low incidence of new fractures during follow-up.

***Summary*:**

Fracture Liaison Services programs reduce mortality and the risk of refracture. A retrospective follow-up study of a cohort of patients with vertebral and nonvertebral fractures managed in a Fracture Liaison Services, showed that the effectiveness was 73.6%. On follow-up, 9.1% of patients had experienced a new fall, and of those 3.7% presented a new fracture.

**Supplementary information:**

The online version contains supplementary material available at 10.1007/s11657-023-01331-w.

## Introduction

Osteoporosis is a systemic skeletal disease characterized by reduced bone mass and impaired bone microarchitecture that predisposes patients to an increased risk of fractures, even with low-energy trauma [1, 2]. These fractures are known as fragility fractures and can be located in the hip, spine, wrist, humerus, tibia and pelvis [1, 3]. Osteoporosis and fractures are very common. Thus, it was estimated that by 2019, there were 178 million new fractures in the world, representing an increase of 33.4% since 1990 [4]. Fragility fractures are associated with high disability, loss of independence, reduced quality of life for patients and caregivers, and high costs for health care systems [2, 3, 5]. Despite the associated high rates of morbidity and mortality, osteoporosis continues to be underdiagnosed and undertreated [6]. Treatment of osteoporosis after a fragility fracture reduces the risk of new fractures [3, 5].

Fracture Liaison Services are programs that provide secondary prevention for fragility fractures [5]. They identify potential patients, evaluate their risk of fractures (by means of bone mineral densitometry, spinal radiographs, and clinical examinations), intervene (using anti-osteoporotic medications, exercise, fall prevention education, etc.) and monitor those who have suffered fractures to prevent new events [7, 8]. Fracture Liaison Services can be evaluated to determine their effectiveness. The indicators used vary widely and may involve identification, diagnosis, interventions, and/or clinically relevant long-term outcomes, including mortality and readmission rates [7]. Recently, 11 key performance indicators were published that seek to evaluate the performance of various fracture liaison services [9]. These key performance indicators are recommended by the International Osteoporosis Foundation (IOF)'s Capture the Fracture® Campaign, the Fragility Fracture Network (FFN) and the Bone Health and Osteoporosis Foundation (BHOF) [9] and are implemented in different countries [7].

The Colombian Health System offers universal coverage to the entire population through two affiliated regimes: the contributory regime that is paid by workers and employers and the subsidized regime that is responsible for the insurance of all people without the ability to pay. This latter plan provides coverage for the surgical and medical management of fractures, as well as specific medications approved for the management of osteoporosis. Although the evidence shows that Fracture Liaison Services programs reduce mortality and the risk of refractures and increase treatment and adherence rates [1, 5, 8], there are few studies published on postfracture care programs in the country, and there is no information on their effectiveness [10, 11]. Consequently, the present study aims to determine the characteristics of patients over 50 years old who suffered fractures and the effectiveness of a Fracture Liaison Services program in a health care institution in Colombia.

## Materials and methods

### Study design and patients

A retrospective study and follow-up of a cohort of patients with vertebral and nonvertebral fractures was carried out, by reviewing medical charts. All of the subjects were treated in a High-Complexity University Hospital in Colombia, which has a Fracture Liaison Services program. The hospital serves patients who subscribe to both the contributory insurance regime (95%) and the state subsidized regime (5%). The program is coordinated by a geriatrician and has a multidisciplinary team made up of orthopedics, pain medicine, physiotherapy and nursing. The program is dedicated to identifying, investigating, intervening and monitoring patients with fractures. Patients are identified through interconsultations made to orthogeriatrics, reports from the diagnostic imaging service, and active search for cases, in the emergency services, hospitalization or outpatient consultation. Follow-up is carried out through face-to-face consultations and by telephone. All patients with a confirmed diagnosis of fracture, of either sex and age 50 or older, and with at least one outpatient follow-up after hospital discharge were eligible in the period between January 1, 2019, and December 31, 2022. Those with high-impact fractures secondary to traffic accidents and patients with total and severe dependence based on a Barthel Index score < 40 points were excluded. The cutoff point for follow-up was February 15, 2023, Therefore, the follow-up time could be variable for each of the patients.

### Variables

Based on the information from the medical records and the Fracture Liaison Services program, a database was designed that allowed the following groups of variables to be collected from the patients:

1. Sociodemographic: sex, age, marital status, origin (urban, rural), contributory or subsidized insurance
2. Clinical: anatomical site of the fracture (hip, spine, femur, humerus, radius/ulna, tibia/fibula, costal, others), number of vertebral fractures, degree of vertebral fracture (mild, moderate, severe), type of fracture (symptomatic or nonsymptomatic), mechanism of fracture (accidental, dizziness/syncope, slipping, seizure, instability, stumbling, etc.), complications (anemia, heart failure, infections, others), history of fragility fractures, index of Charlson comorbidities, Barthel Index (total dependence, < 20 points; severe dependence, 20–35 points; moderate dependence:, 40–60 points; mild dependence, 65–85 points; and independence, 90–100 points), polypharmacy (5 or more medications), smoking (past, active), alcohol intake, weight, height and body mass index (BMI).
3. Paraclinical (on admission): hemoglobin, alkaline phosphatase, thyroid stimulating hormone, albumin, corrected calcium (calcium by colorimetry adjusted to albumin), parathyroid hormone, vitamin D, testosterone, creatinine (glomerular filtration rate was calculated by the CDK EPI 2021 equation) and bone mineral densitometry (T score of the femoral and vertebral neck, Fracture Risk Assessment Tool (FRAX) of the hip and major osteoporotic fracture). According to the cut-off points of the reference clinical laboratory, albumin levels were determined as hypoalbuminemia. < 3.5 g/dL, hypocalcemia < 8.5 mg/dL, hyperparathyroidism > 65.0 pg/mL and vitamin D deficiency/insufficiency < 30.0 ng/mL.
4. Management: prior therapy for osteoporosis (bisphosphonates, denosumab, teriparatide, others), prior calcium/vitamin D replacement, surgical intervention (osteosynthesis, joint replacement, etc.), walking rehabilitation in hip fractures (< 24 h, 24–48 h, > 48 h), antiosteoporotic therapy at discharge (bisphosphonates, denosumab, teriparatide, others), calcium/vitamin D replacement at discharge, and thromboprophylaxis at discharge (heparins, direct oral anticoagulants, others).
5. Monitoring (controls): Barthel Index, Functional Ambulation Categories scale (FAC) (level 0, no ambulation; level 1, functional ambulation; level 2, home ambulation; level 3, ambulation around the house or neighborhood; level 4, independent ambulation in the community; level 5, normal ambulation), calcium, parathyroid hormone, vitamin D, bone mineral densitometry, antiosteoporotic therapy, calcium/vitamin D replacement, new falls, new fractures and vital status (alive or dead).
6. Effectiveness of the Fracture Liaison Services: evaluated using the 11 Key Performance Indicators [9]. The effectiveness of the program was evaluated in those patients who had a follow-up of 52 weeks or more.

### Ethical statement

The protocol was endorsed by the Bioethics Committee of the Technological University of Pereira in the category of "research without risk" (approval code: 78–311 022). The principles of confidentiality of information established by the Declaration of Helsinki were respected.

### Data analysis

The data were analyzed with the statistical package SPSS Statistics, version 26.0 for Windows (IBM, USA). A descriptive analysis was carried out with frequencies and proportions for the qualitative variables and measures of central tendency and dispersion for the quantitative variables by means and standard deviation. The comparison of quantitative variables was carried out using Student's t test and X ^2^ or Fisher's exact test for categorical variables. A level of statistical significance was established at p < 0.05.

## Results

### Sociodemographic

A total of 752 patients had fractures during the study period and 438 (58.2%) patients who met the inclusion criteria were analyzed. A total of 78.5% (*n* = 344) were women, and the average age was 77.5 ± 9.5 years (range: 53.0–99.0 years). Most subject (89.3%, *n* = 391) were 65 years of age or older, did not have a partner (55.3%, *n* = 242), came from urban areas (89.3%, *n* = 391) and the most common insurance scheme was contributory (97.0%, *n* = 425) (Table 1).

**Table 1:** Sociodemographic and clinics characteristics of a group of patients treated in a Fracture Liaison Services program, in a highly complex clinic, Colombia

Variables	Total		Women		Men		*p*
	*n* = 438	%	*n* = 344	%	*n* = 94	%	
Age (years), mean (SD)	77.5 ± 9.5		77.6 ± 9.5		77.1 ± 9.6		0.621
50–64 years	47	10.7	35	10.2	12	12.8	0.472
65–74 years	122	27.9	97	28.2	25	26.6	0.759
75–84 years	153	34.9	119	34.6	34	36.2	0.776
≥ 85 years	116	26.5	93	27.0	23	24.5	0.617
Marital status	-	-	-	-	-	-	-
Married	187	42.7	127	36.9	60	63.8	< 0.001
Widower	135	30.7	126	36.6	9	9.6	< 0.001
Single	84	19.2	70	20.3	14	14.9	0.234
Separate	23	5.3	17	4.9	6	6.4	0.579
Relationship	9	2.1	4	1.2	5	5.3	0.025
Origin	-	-	-	-	-	-	-
Urban	391	89.3	307	89.2	84	89.4	0.974
Rural	47	10.7	37	10.8	10	10.6	
Affiliation regime	-	-	-	-	-	-	-
Contributory	425	97.0	333	96.8	92	97.9	0.588
Subsidized	13	3.0	11	3.2	2	2.1	
Charlson Comorbidity Index (points), mean (SD)	4.1 ± 1.9		4.0 ± 1.9		4.3 ± 2.1		0.292
≥ 3 points	309	70.5	242	70.3	67	71.3	0.861
Barthel index (points), mean (SD)	94.4 ± 12.6		94.1 ± 12.8		94.8 ± 12.8		0.536
Anthropometric measures	-	-	-	-	-	-	-
Weight (Kg), mean (SD)	61.8 ± 11.5		60.7 ± 11.4		66.2 ± 11.2		< 0.001
Body mass index (Kg/m2), mean (SD)	25.7 ± 4.1		26.0 ± 4.2		24.6 ± 3.1		0.001
Fracture site	-	-	-	-	-	-	-
Hip	111	25.3	86	25.0	25	26.6	0.753
Vertebral	109	24.9	78	22.7	31	33.0	0.041
Radius/ulna	80	18.3	70	20.3	10	10.6	0.031
Humerus	69	15.7	52	15.1	17	18.1	0.484
Tibia/fibula	23	5.3	20	5.8	3	3.2	0.436
Pelvis	17	3.9	15	4.4	2	2.1	0.545
Other	29	6.6	23	6.7	6	6.4	0.917
Fracture type	-	-	-	-	-	-	-
Symptomatic	348	79.5	286	83.1	62	66.0	< 0.001
Not symptomatic	90	20.5	58	16.9	32	34.0	
Fracture mechanism	-	-	-	-	-	-	-
Slipping	113	25.8	95	27.6	18	19.1	0.096
Tripping	101	23.1	83	24.1	18	19.1	0.310
No trauma	83	18.8	59	17.2	24	25.5	0.066
Instability	63	14.4	49	14.2	14	14.9	0.874
Dizziness/syncope	16	3.7	10	2.9	6	6.4	0.123
Accidental	13	3.0	10	2.9	3	3.2	1,000
Others (*n* = 6)	15	3.4	11	3.2	4	4.3	0.539
Unknown	34	7.8	27	7.8	7	7.4	0.897

*SD*: Standard Deviation

### Clinicians

The mean Barthel Index was 94.4 ± 12.6 points (range: 40–100), and the majority of subjects were considered to be functionally independent (83.6%, *n* = 366). The average Charlson Comorbidities Index was 4.1 ± 1.9 (range: 1–9), and 29.7% (*n* = 130) had a report of a previous fracture. A total of 5.3% (*n* = 23) were active smokers, and 2.5% (*n* = 11) consumed alcohol. The most common fractures were hip fractures, and the majority of patients had a walking time of less than 48 h (76.6%, *n* = 85/111), followed by vertebral fractures with an average of 1.8 ± 1. 1 fractures, of which the majority were severe (43.1%, *n* = 47/109). The proportion of vertebral fractures was significantly higher in men while radius/ulna fractures were significantly higher in women. Slipping and tripping were the most common mechanisms related to falls. The other clinical variables can be seen in Table 1.

### Paraclinical

The hemoglobin of the patients had a reduction of 1.6 g/dL (admission: 12.7 g/dL vs. discharge; 11.1 g/dL; *p* =  < 0.001) during hospitalization (Table 2). In the subgroup of patients with hip fracture, the reduction in hemoglobin was greater (difference of means: 2.4 g/dL; 12.7 g/dL vs. 10.3 g/dL; *p* =  < 0.001) compared to the other types of fractures (Supplementary Table 1). At the time of admission, a total of 35.2% (*n* = 154) of the patients had hypocalcemia, 35.6% (*n* = 156) had hyperparathyroidism, and 64.6% (*n* = 283) had vitamin D deficiency or insufficiency. Hypoalbuminemia was found in 31.1% (*n* = 136) of the cases. A total of 18.9% (*n* = 83) had a glomerular filtration rate < 60 ml/min/1.73 m^2^. Previous bone mineral densitometry was reported in 15.3% (*n* = 67) of the patients (osteoporosis 10.7% and osteopenia 4.6%). Bone mineral densitometry performed after the fracture was reported in 70.8% (*n* = 310) of the patients and revealed osteoporosis in 44.5% (*n* = 195) and osteopenia in 21.9% (*n* = 96) of the cases. There were no significant differences in the proportion of women and men with bone mineral densitometry, but the parameters evaluated were generally worse in women (Table 2 and Supplementary Table 1).

**Table 2:** Paraclinical characteristics of a group of patients treated in a Fracture Liaison Services program, in a highly complex clinic, Colombia

Variables	Total		Women		Men		*p*
	*n* = 438	%	*n* = 344	%	*n* = 94	%	
Baseline clinical laboratory (admission)	-	-	-		-		-
Admission hemoglobin (g/dL), mean (SD); *n* = 354	12.7 ± 1.8		12.5 ± 1.7		13.1 ± 2.2		0.051
Discharge hemoglobin (g/dL), mean (SD); *n* = 294	11.1 ± 1.9		11.0 ± 1.7		11.5 ± 2.3		0.125
Alkaline phosphatase, mean (SD); *n* = 317	99.1 ± 141.1		93.6 ± 49.4		119.1 ± 288.4		0.467
TSH (uIU/mL), mean (SD); *n* = 337	3.4 ± 4.0		3.2 ± 3.3		4.3 ± 5.6		0.111
Albumin (g/dL), mean (SD); *n* = 328	3.5 ± 0.4		3.5 ± 0.4		3.5 ± 0.5		0.810
Calcium (mg/dL), mean (SD); *n* = 336	8.6 ± 0.6		8.7 ± 0.6		8.6 ± 0.7		0.280
PTH (pg/mL), mean (SD); *n* = 342	69.1 ± 48.4		70.0 ± 52.9		66.0 ± 26.6		0.523
Vitamin D (ng/mL), mean (SD); *n* = 344	22.2 ± 8.9		21.8 ± 9.2		23.7 ± 8.0		0.104
Testosterone (ng/mL), mean (SD); *n* = 59	3.7 ± 2.4		-		3.7 ± 2.4		-
Creatinine (mg/dL), mean (SD); *n* = 352	0.9 ± 0.5		0.8 ± 0.3		1.2 ± 0.8		< 0.001
GFR (mL/min/1.73 m^2^), mean (SD)	74.2 ± 19.8		75.0 ± 18.8		71.5 ± 22.6		0.201
Bone mineral densitometry before index fracture	67	15.3	55	16.0	12	12.8	0.442
Vertebral BMD -T sore—(points), mean (SD); *n* = 67	-2.5 ± 1.4		-2.4 ± 1.2		-2.1 ± 0.9		0.590
Femoral neck BMD -T score- (points), mean (SD); *n* = 67	-2.5 ± 0.9		-2.4 ± 0.8		-2.0 ± 1.1		0.237
FRAX probability for hip fracture (%), mean (SD); *n* = 39	3.2 ± 2.9		3.5 ± 2.9		1.0 ± 0.7		0.107
FRAX probability for MOF (%), mean (SD); *n* = 39	8.1 ± 5.2		8.6 ± 5.2		3.2 ± 0.8		0.044
Bone mineral densitometry after index fracture	310	70.8	247	71.8	63	67.0	0.366
Vertebral BMD -T sore—(points), mean (SD); *n* = 307	-2.1 ± 1.6		-2.3 ± 1.6		-1.4 ± 1.6		< 0.001
Femoral neck BMD -T score- (points), mean (SD); *n* = 308	-2.4 ± 1.2		-2.4 ± 1.3		-2.3 ± 1.1		0.418
FRAX probability for hip fracture (%), mean (SD); *n* = 246	3.5 ± 3.1		3.8 ± 3.3		2.4 ± 1.7		< 0.001
FRAX probability for MOF (%), mean (SD); *n* = 246	8.3 ± 4.8		9.1 ± 4.9		5.0 ± 2.7		< 0.001

*SD*: Standard Deviation; *TSH*: Thyroid-Stimulating Hormone; *PTH*: Parathormone; *GFR*: Glomerular Filtration Rate; *BMD*: Bone Mineral Density; *FRAX*: Fracture Risk Assessment Tool; MOF major osteoporosis fracture (low trauma fractures of the hip. clinical spine. wrist and humerus); FRAX 10-year fracture risk probabilities

### Treatment

Polypharmacy was found in 46.8% (*n* = 205) of the patients. A total of 21.7% (*n* = 95) were receiving calcium/vitamin D supplementation, and 16.7% (*n* = 73) had been prescribed antiosteoporotic therapy before the index fracture. Most of the patients had inadequate dairy intake (73.7%, *n* = 323). A total of 63.5% (*n* = 278) of the patients received surgical management, and 3.4% (*n* = 15) required transfer to the intensive care unit. Surgical management predominated in patients with hip fractures. Most of them were discharged with a calcium/vitamin D prescription (58.7%, *n* = 257; women 58.4% vs men 59.6%, *p* = 0.842), and half were discharged with an indication for antiosteoporotic therapy (50.7%, *n* = 222; women 51.7% vs men 46.8%, *p* = 0.396). Enoxaparin was the most widely used anticoagulant (24.9%, *n* = 109). There were no in-hospital deaths. Antiosteoporotic treatment overall was significantly more prevalent in women with hip fractures compared to men (83.7% vs 64.0; *p* = 0.032). No other differences were found (Table 3).

**Table 3:** Management of a group of patients treated in a Fracture Liaison Services program, in a highly complex clinic, Colombia

Variables	Total		Women		Men		*p*
	*n* = 438	%	*n* = 344	%	*n* = 94	%	
Hip fracture	111	25.3	86	25.0	25	26.6	0.753
Surgical management	104	93.7	81	94.2	23	92.0	0.692
Osteosynthesis	79	71.2	61	70.9	18	72.0	0.917
Joint replacement	25	22.5	20	23.3	5	20.0	0.732
Pharmacological management at discharge	-	-	-	-	-	-	-
Calcium/vitamin D supplementation	89	80.2	69	80.2	20	80.0	0.980
Antiosteoporotic treatment	88	79.3	72	83.7	16	64.0	0.032
Denosumab	37	33.3	30	34.9	7	28.0	0.520
Teriparatide	30	27.0	25	29.1	5	20.0	0.369
Zoledronic acid	20	18.0	16	18.6	4	16.0	1,000
Oral bisphosphonate	1	0.9	1	1.2	0	0.0	1,000
Thromboprophylactic therapy	93	83.8	72	83.7	21	84.0	0.973
Vertebral fracture	109	24.9	78	22.7	31	33.0	0.041
Surgical management	14	12.8	9	11.5	5	16.1	0.535
Vertebroblasty	13	11.9	8	10.3	5	16.1	0.513
Laminectomy	1	0.9	1	1.3	0	0.0	1,000
Pharmacological management at discharge	-	-	-	-	-	-	-
Calcium/vitamin D supplementation	58	53.2	43	55.1	15	48.4	0.525
Antiosteoporotic treatment	66	60.6	50	64.1	16	51.6	0.229
Teriparatide	41	37.6	33	42.3	8	25.8	0.109
Zoledronic acid	17	15.6	9	11.5	8	25.8	0.064
Denosumab	8	7.3	8	10.3	0	0.0	0.102
Thromboprophylactic therapy at discharge	3	2.8	1	1.3	2	6.5	0.194
Others fractures	218	49.8	180	52.3	38	40.4	0.041
Surgical management	155	71.1	130	72.2	25	65.8	0.427
Osteosynthesis	145	66.5	120	66.7	25	65.8	0.917
Joint replacement	10	4.6	10	5.6	0	0.0	0.216
Pharmacological management at discharge	-	-	-	-	-	-	-
Calcium/vitamin D supplementation	110	50.5	89	49.4	21	55.3	0.514
Antiosteoporotic treatment	68	31.2	56	31.1	12	31.6	0.955
Denosumab	27	12.4	21	11.7	6	15.8	0.587
Teriparatide	22	10.1	18	10.0	4	10.5	1,000
Zoledronic acid	17	7.8	15	8.3	2	5.3	0.743
Oral bisphosphonate	2	0.9	2	1.1	0	0.0	1,000
Thromboprophylactic therapy at discharge	27	12.4	24	13.3	3	7.9	0.430

### Follow-up

A total of 318 patients (72.6%) had a follow-up of 52 or more weeks. All patients had a control visit, 57.3% (*n* = 251) had two visits, 38.8% (*n* = 170) received three visits, and 24.9% (*n* = 109) had four visits. The first medical check-up was conducted an average of 83 days after discharge. A total of 89.1% (*n* = 390) of the patients had a level 3 or higher on the functional gait rating scale. Most were receiving calcium/vitamin D supplementation (74.9%, *n* = 328) and antiosteoporotic therapy (61.9%, *n* = 271), especially with teriparatide (23.7%, *n* = 104). Table 4 shows the visits of the patients treated in the Fracture Liaison Services program. In the follow-up of 682.9 ± 392.6 days, 9.1% (*n* = 40) had new falls, 3.7% (*n* = 16) had new fractures (especially vertebral *n* = 6) and 4.3% (*n* = 19) had died on average 406 ± 302.9 days after discharge.

**Table 4:** Visits of a group of patients treated in a Fracture Liaison Services program, in a high complexity clinic, Colombia

Variables	Visit 1		Visit 2		Visit 3		Visit 4	
	*n* = 438	%	*n* = 251	%	*n* = 170	%	*n* = 109	%
Time between discharge and control (days), mean (SD)	83.0 ± 143.6		228.1 ± 175.8		407.2 ± 165.8		577.6 ± 141.7	
Calcium/vitamin D supplementation	328	74.9	225	89.6	158	92.4	102	93.6
Antiosteoporotic therapy	271	61.9	222	88.4	163	95.9	106	97.2
Teriparatide	104	23.7	96	38.2	77	45.3	53	48.6
Denosumab	85	19.4	67	26.7	46	27.1	31	28.4
Zoledronic acid	75	17.1	56	22.3	36	21.2	21	19.3
Oral bisphosphonate	6	1.4	2	0.8	3	1.8	1	0.9
Romozosumab	1	0.2	1	0.4	1	0.6	0	0.0
None	165	37.6	29	11.6	7	4.1	3	2.8
Barthel index (points), mean (SD)	94.1 ± 12.5		94.8 ± 11.2		94.6 ± 13.0		96.3 ± 10.1	
Independence (90–100 points)	361	82.5	208	82.9	144	84.7	97	89.0
Mild dependency (65–85 points)	51	11.6	32	12.7	15	8.8	8	7.3
Moderate dependency (40–60 points)	21	4.8	10	4.0	10	5.8	4	3.7
Unrealized	5	1.1	1	0.4	1	0.6	0	0.0
Functional Ambulation Categories (FAC) Scale	3.9 ± 0.9		4.0 ± 0.9		4.0 ± 0.9		4.1 ± 0.9	
No wandering (level 0)	0	0.0	0	0.0	0	0.0	0	0.0
Functional ambulation (level 1)	1	0.2	0	0.0	0	0.0	0	0.0
Ambulation at home (level 2)	34	7.8	18	7.2	9	5.3	3	2.8
Walking around the house or neighborhood (level 3)	112	25.6	66	26.3	41	24.1	26	23.9
Independent ambulation in the community (level 4)	134	30.6	76	30.3	58	34.1	37	33.9
Normal walking (level 5)	144	32.9	89	35.5	61	35.8	43	39.4
Unrealized	13	2.9	2	0.7	1	0.6	0	0.0
New fracture	8	1.8	4	1.6	2	1.2	2	1.8
PTH (pg/mL), mean (SD)	64.6 ± 43.4		65.0 ± 37.7		52.7 ± 25.7		58.6 ± 31.2	
Normal (15.0–65.0 pg/mL)	188	42.9	85	33.9	74	43.5	54	49.5
High (> 65.0 pg/mL)	131	29.9	26	10.4	22	12.9	17	15.6
Low (< 15.0 pg/mL)	1	0.2	7	2.8	2	1.2	1	0.9
Unrealized	118	26.9	133	52.9	72	42.4	37	33.9
Vitamin D (ng/mL), mean (SD)	23.1 ± 9.6		26.2 ± 9.2		28.0 ± 12.0		27.4 ± 13.2	
Normal (30.0–60.0 ng/mL)	63	14.3	21	8.4	26	15.3	13	11.9
Deficiency (< 21.0 ng/mL)	140	32.0	54	21.5	33	19.4	25	22.9
Insufficiency (21.0–29.9 ng/mL)	116	26.5	47	18.7	40	23.5	34	31.2
Unrealized	119	27.2	129	51.4	71	41.8	37	33.9
Calcium (mg/dL), mean (SD)	8.9 ± 0.9		9.5 ± 0.5		9.8 ± 0.6		9.0 ± 0.0	
Normal (8.6–10.2 mg/dL)	200	45.7	112	44.6	91	53.5	71	65.1
Low (< 8.5 mg/dL)	90	20.5	8	3.2	3	1.8	0	0.0
High (> 10.2 mg/dL)	8	1.8	10	4.0	10	5.9	4	3.7
Unrealized	140	32.0	121	48.2	66	38.8	34	31.2

*SD*: Standard Deviation; PTH: Parathormone

### Effectiveness

At one year, the average effectiveness of the Fracture Liaison Services program was 73.6%. For at least five indicators, effectiveness was equal to or greater than 80%, and only one key performance indicator was less than 50% (Table 5).

**Table 5:** Effectiveness of a Fracture Liaison Services program, in a highly complex clinic, Colombia

Key Performance Indicators	%	Level of achivement
Indicator 1: Identification of patients with non-spine fragility fractures	56.6	Average
Indicator 2: Identification patients with spine fractures	78.4	Average
Indicator 3: Initial investigation including fracture risk assessment within 12 weeks	79.2	Average
Indicator 4: Dual x-ray absorptiometry (DXA) within 12 weeks	55.2	Average
Indicator 5: Falls risk assessment	97.8	Good
Indicator 6: Anti-osteoporosis medication (AOM) recommended as appropriate	99.4	Good
Indicator 7: Recorded follow-up within 16 weeks post index fracture	88.7	Good
Indicator 8: Commenced anti-osteoporosis medication (AOM) by 16 weeks post index fracture	52.1	Average
Indicator 9: Strength and balance commenced within 16 weeks of fracture	28.1	Poor
Indicator 10: Patients taking anti-osteoporosis medication 52 weeks after the sentinel fracture	84.4	Good
Indicator 11: Data completeness	90.0	Good

## Discussion

This study allowed us to characterize the sociodemographic, clinical, paraclinical and pharmacological variables of a group of patients who presented fractures treated in a Fracture Liaison Services program and to evaluate the program’s effectiveness using key performance indicators. Hip fractures were the most prevalent in this program without finding significant differences according to sex, while vertebral fractures predominated in men. No major differences were found in sociodemographic variables and in surgical and pharmacological management between women and men; but women with hip fractures were more often treated with anti-osteoporotic drugs. Most of the patients had a follow-up of more than 52 weeks and the effectiveness of the program in this group of patients was high. Few patients presented a new fracture during follow-up. The approach to this topic is very relevant because the population is aging, and it is the elderly who are at a greater risk of fractures [1, 4]. Access to injury prevention programs and access to osteoporosis detection and treatment should help reduce morbidity and mortality caused by fragility fractures [4]. Although Fracture Liaison Services programs have been established in many parts of the world, they serve a minority of people, so broader coverage should be a priority [1]. Knowing the characteristics and effectiveness of these programs can encourage their implementation in a greater number of care centers in the country and worldwide.

The median age of the patients was similar to that found in other studies (75.3–79.0 years) [10–17], with a predominance of women, as identified by previous studies (71.0–91.1%) [10–15, 17–26]. Similarly, the Charlson Comorbidities Index of these patients was high, as described in the literature [12, 15, 19]. A total of 29.7% had a history of fractures, which is consistent with what was found in a Colombian study (27.3%) [10] and contrasts with what was found in European countries (45.9%-53.0%) [15, 19–21] and South America (39.1%-40.0%) [11, 22]. These conditions have been considered risk factors for the appearance of new fragility fractures. [2]. Women and men with a previous fragility fracture should be considered as a priority to start a pharmacological intervention due to the high risk of subsequent fractures. [2]. However, only 16.7% of the patients were receiving an antiosteoporotic drug, showing poor coverage as previously described in the country (7.0%-12.1%) [10, 11, 17, 27] and in the world (10.8%-21.3%) [15, 18–22, 24].

Hip fractures were the most frequent fracture, which is consistent with other studies conducted in Colombia (36.7%-58.2%) [10, 11, 26], Spain (33.6% -45.0%) [23, 24] and the United States (35.3%) [28]. It is important to highlight that vertebral fractures were found in almost a quarter of the patients, similar to the findings of other reports (22.6%-28.1%) [10, 22, 23] but in contrast with other research (39.0%-64.2%) [13–15, 18, 19]. It has been reported in the literature that the anatomical location of fractures may differ between women and men. [29]. In this report, it was found that vertebral fractures prevailed in men while radius/ulna fractures prevailed in women. Sex differences in the type of fractures may be related to body and bone size, bone mineral density, and hormonal changes. [30]. Half of the patients reported a slip or stumble as a mechanism that led to the fall and fracture. It is striking that the cause of falls is not reported in studies on postfracture care programs [10–15, 18–25]. By recognizing the mechanisms of falls, multifactorial interventions can be improved and individualized to prevent new falls, for example, balance training and evaluation and modification of the environment [31, 32].

Vitamin D deficiency or insufficiency was found in two-thirds of the patients, which is consistent with reports from Spain (81.4%) [25], Israel (75.0%) [16] and Brazil (55.5%) [22] but much higher than that described in Europe (6.8% -35.9%) [20, 21] and the United States (18.0%) [18] and in a previous study in Colombia (22.9%) [11]. In addition, slightly more than a third of the patients had hyperparathyroidism or hypocalcemia, which is higher than that reported in Israel (25% and 22%, respectively) [16]. Hyperparathyroidism, calcium deficiency and vitamin D deficiency are risk factors that contribute to the development of secondary osteoporosis [31, 33]. The correction of these deficiencies, ensuring a total daily intake of calcium of 800 to 1200 mg and a serum level of 25-hydroxyvitamin D of at least 40 ng/mL, is recommended by the local clinical practice guidelines [6]. Additionally, it has been documented that the parameters evaluated in bone mineral densitometry tend to be worse in women compared to men [34], which is also consistent with what was found in this report. Estrogen deficiency contributes to osteoporosis in both sexes, and it is more significant in women and begins at a younger age [2].

Most of the patients were managed surgically and with placement of osteosynthesis material, as evidenced in the literature [10, 11, 17, 20]. A total of 58.7% of the patients were prescribed calcium/vitamin D supplementation at discharge, which is consistent with other reports (62.1%-63.1%) [10, 20]. Half of the patients were prescribed antiosteoporotic medication at discharge, which was higher than that reported in other programs in Colombia (39.4%-42.4%) [10, 11] and in the world (13.4%-37.8%) [12, 20]. However, the prescription was higher in France (94.9%-96.6%) [15, 19], the United States (94.9%) [28], Spain (74.0%-78.4%) [24, 25] and Israel (73%) [16]. It is important to highlight that two studies in Columbia reported a notoriously lower use of calcium/vitamin D and antiosteoporotic drugs in care centers that did not have Fracture Liaison Services programs [17, 26]. The use of bisphosphonates, teriparatide, and denosumab, among others, to varying degrees, has been related to reducing the risk of new vertebral and nonvertebral fractures [6, 33]. In this report, pharmacological management did not differ between women and men, which contrasts with a pharmacoepidemiological study carried out in Colombia, where it was significantly evidenced that women were treated in a higher proportion with anti-osteoporotic drugs and with calcium and vitamin D supplements compared to men [27].

The most prescribed antiosteoporotic drug at the time of discharge and in outpatient check-ups was teriparatide, which is consistent with what was reported in a local study (26.4%) [11] and in the United States (56.5%) [28]. Less use has been evidenced in European countries (1.0%-16.0%) [19, 20, 23, 25] and Asia (1.3%-9.0%) [12, 16]. Bisphosphonates predominate in countries such as Spain (62.6%-81.8%) [23–25], France (62.9%-65.5%) [15, 19] and Thailand (26.7%) [12], while denosumab has been the most commonly used medication in Israel (57.0%) [16], Brazil (54.1%) [22] and Finland (47.5%) [20]. Variation in drug use patterns may be due to different causes, including the preferences of the prescribing physician, the severity of osteoporosis, the availability of the drug, contraindications and the indications provided by the clinical practice guidelines [27]. In this sense, local recommendations indicate that patients with fragility fractures should be treated with denosumab or teriparatide, considering their high risk of developing new fractures. In patients with clinical conditions that contraindicate these two drugs, zoledronic acid is an option [6]. The foregoing evidence supports the adequate adherence of the program to the clinical practice guidelines.

The average effectiveness of the fracture program was 73.6%, with a single indicator below 50%. Two publications were found on postfracture care programs in the country, but neither of them evaluated their effectiveness [10, 11]. In England and Wales, in 2020, 62,207 patients who received services in 69 Fracture Liaison Services programs were evaluated and an overall effectiveness of 38.5% was found with 7 indicators below 50% [35]. Fracture Liaison Services were affected by the COVID-19 pandemic [35]. In another study, the effectiveness was evaluated using the International Osteoporosis Foundation Quality Standards, evidencing an average effectiveness of 78.5% with only two indicators below 50% in a fracture program in Spain [23]. Although the overall effectiveness of this program was satisfactory, it is necessary to implement an improvement plan that addresses the indicator related to the prevention of falls by accepting the recommendations of different guides [9, 31, 32].

A total of 9.0% of subjects reported a new fall at follow-up, which was consistent with what was found in the follow-up of a group of patients treated in a fracture care program in Colombia (5.8%) [10] and is notably lower than that found in a study in Taiwan (33.1%) [14]. According to the FRAX® T score, the probability of hip fractures and major osteoporotic fractures was lower in this study than in other investigations [13, 14, 19, 24]. Thus, 3.7% presented a new fracture, which is in line with other reports (1.0%-4.0%) [10, 12, 13, 28] and with proportions lower than those found in Spain (6.6% -15.2%) [24, 25], Brazil (11.1%) [22], the United States (8.4%) [18] and Taiwan (6.0%) [14]. Fracture Liaison Services programs significantly improved the proportion of patients with bone mineral densitometry, initiation of treatment, and adherence to management and reduced the incidence of new fractures and mortality [8].

Since 2012, the Capture the Fracture initiative began supporting fracture secondary prevention services around the world. The use of the resources of the Capture the Fracture initiative, including Fracture Liaison Services programs workshops with direct support to groups of coordinators and mentoring with experts worldwide, led to the mapping of these programs [36]. In 2021, three specialist doctors from Colombia were trained through a mentoring program with the support of the IOF and the University of Oxford. This has allowed the development and generation of resources with a view to optimizing the implementation in the country of post-fracture care models. In addition, with the support of the Colombian Association of Osteoporosis and Mineral Metabolism, the training of health professionals at the national level has continued [36, 37]. This is how in the last 2 years there has been an increase in the number of institutions, both public and private, that have adopted the framework of good practices of the IOF, and have been implementing the 11 Key Performance Indicators [36]. The first Colombian hip fracture registry is currently being developed, to be carried out with all the centers identified in the IOF good practice map and based on the variables of the multiple existing European registries [36, 38].

Some limitations should be considered when interpreting the results. The data were obtained from a group of patients from a health provider institution, mainly from the contributory regime of the Colombian health system, so the findings may not be extrapolated to other insurers. In addition, for a few variables, information was not available for all patients because the data obtained from individual medical records was incomplete. It was not possible to evaluate the effectiveness of the program for all the patients because the follow-up in 27.4% of the cases was less than 52 weeks. However, the study included a significant number of men and women of different age groups and with different types of fragility fractures. In addition, it is the first report that evaluates the effectiveness of a Fracture Liaison Services program in the country.

## Conclusions

With the findings identified, it can be concluded that this group of patients who suffered fractures and were cared for by the Fracture Liaison Services program who commonly presented risk factors for a new fragility fracture such as advanced age, female sex, osteoporosis, other comorbidities and previous fractures. The anatomical location of the fracture differed by sex and there were no significant differences in terms of treatment, except in women with hip fractures who received more anti-osteoporotic drugs.. Adherence to the recommendations of the country's clinical practice guidelines was significant in their recovery. Overall, the results indicate that the program was very effective, and there was a low incidence of new fractures during follow-up. Greater implementation of Fracture Liaison Services programs in the country should be promoted to improve the coverage and quality of care for patients with fragility fractures.

### Supplementary information

Below is the link to the electronic supplementary material.

Supplementary file1 (DOCX 16 KB)

## Data Availability

Protocols.io.

## Declarations

### Data access

https://www.protocols.io/private/6A1CE45DE2EC11ED813C0A58A9FEAC02.
