# Supplementary material for: Characterization and effectiveness of a Fracture Liaison Services program in Colombia
Source: Arch Osteoporos. 2023 Oct 3;18(1):124. doi: 10.1007/s11657-023-01331-w (PMC10547802; doi:10.1007/s11657-023-01331-w)
Supplement: Supplementary file 1 — Supplementary file1 (DOCX 16 KB) [file 11657_2023_1331_MOESM1_ESM.docx]

**Supplementary Table 1**. Sociodemographic, clinical and paraclinical characteristics of a group of patients treated in a Fracture Liaison Services program, in a highly complex clinic, Colombia.

| **Variables** | **Hip fracture** | | **Vertebral fracture** | | **Others fractures** | |
| --- | --- | --- | --- | --- | --- | --- |
|  | **n=111** | **%** | **n=109** | **%** | **n=218** | **%** |
| Women | 86 | 77.5 | 78 | 71.6 | 180 | 82.6 |
| Age (years), mean (SD) | 80.4 ± 8.2 | | 78.4 ± 8.7 | | 75.6 ± 10.1 | |
| Charlson Comorbidity Index (points), mean (SD) | 4.4 ± 2.0 | | 4.3 ± 1.8 | | 3.8 ± 1.9 | |
| Barthel index (points), mean (SD) | 91.6 ± 13.8 | | 92.4 ± 14.5 | | 96.7 ± 10.7 | |
| Fracture type | - | - | - | - | - | - |
| Symptomatic | 103 | 92.8 | 36 | 33.0 | 209 | 95.9 |
| Not symptomatic | 8 | 7.2 | 73 | 67.0 | 9 | 4.1 |
| Fracture mechanism | - | - | - | - | - | - |
| Slipping | 32 | 28.8 | 16 | 14.7 | 65 | 29.8 |
| Tripping | 32 | 28.8 | 1 | 0.9 | 68 | 31.2 |
| No trauma | 5 | 4.5 | 75 | 68.8 | 3 | 1.4 |
| Instability | 28 | 25.2 | 6 | 5.5 | 29 | 13.3 |
| Dizziness/syncope | 5 | 4.5 | 1 | 0.9 | 10 | 4.6 |
| Accidental | 3 | 2.7 | 2 | 1.8 | 8 | 3.7 |
| Others (n=6) | 1 | 0.9 | 4 | 3.7 | 10 | 4.6 |
| Unknown | 5 | 4.5 | 4 | 3.7 | 25 | 11.5 |
| Baseline clinical laboratory (admission) | - | - | - | - | - | - |
| Admission hemoglobin (g/dL), mean (SD); n=354 | 12.7 ± 1.8 | | 14.0 ± 2.8 | | 13.2 ± 2.5 | |
| Discharge hemoglobin (g/dL), mean (SD); n=294 | 10.3 ± 2.2 | | 13.0 ± 2.3 | | 11.7 ± 2.3 | |
| Alkaline phosphatase, mean (SD); n=317 | 77.1 ± 22.7 | | 92.0 ± 35.1 | | 80.4 ± 23.3 | |
| TSH (uIU/mL), mean (SD); n=337 | 4.7 ± 2.6 | | 3.6 ± 3.4 | | 6.4 ± 11.3 | |
| Albumin (g/dL), mean (SD); n=328 | 3.3 ± 0.3 | | 3.4 ± 0.4 | | 3.4 ± 0.3 | |
| Calcium (mg/dL), mean (SD); n=336 | 8.3 ± 0.7 | | 8.5 ± 0.7 | | 8.6 ± 0.5 | |
| PTH (pg/mL), mean (SD); n=342 | 67.8 ± 23.9 | | 71.4 ± 34.5 | | 56.0 ± 21.1 | |
| Vitamin D (ng/mL), mean (SD); n=344 | 21.9 ± 8.5 | | 23.2 ± 9.2 | | 21.9 ± 9.1 | |
| Testosterone (ng/mL), mean (SD); n=59 | 2.6 ± 1.8 | | 4.0 ± 1.6 | | 4.0 ± 3.1 | |
| Creatinine (mg/dL), mean (SD); n=352 | 1.2 ± 0.5 | | 1.1 ± 0.2 | | 1.1 ± 0.6 | |
| GFR (mL/min/1.73 m²), mean (SD) | 73.4 ± 23.4 | | 74.6 ± 14.3 | | 77.7 ± 22.8 | |
| Bone mineral densitometry after index fracture | - | - | - | - | - | - |
| Vertebral BMD -T sore - (points), mean (SD); n=307 | -2.5 ± 1.4 | | -2.3 ± 1.6 | | -2.0 ± 1.5 | |
| Femoral neck BMD -T score- (points), mean (SD); n=308 | -2.6 ± 1.2 | | -2.5 ± 1.0 | | -2.2 ± 1.2 | |
| FRAX probability for hip fracture (%), mean (SD); n=246 | 3.7 ± 2.9 | | 3.5 ± 3.0 | | 3.5 ± 3.2 | |
| FRAX probability for MOF (%), mean (SD); n=246 | 7.9 ± 4.2 | | 7.8 ± 5.2 | | 8.7 ± 4.8 | |

SD: Standard Deviation; TSH: Thyroid-Stimulating Hormone; PTH: Parathormone; GFR: Glomerular Filtration Rate; BMD: Bone Mineral Density; FRAX: Fracture Risk Assessment Tool; MOF major osteoporosis fracture (low trauma fractures of the hip. clinical spine. wrist and humerus); FRAX 10-year fracture risk probabilities.
